# Optimization of glucocorticoid administration in patients with sepsis using reinforcement learning: a multicenter retrospective study

**DOI:** 10.3389/fcimb.2026.1856196

**Published:** 2026-08-19

**Authors:** Yujing Zhang, Lei Liang, Xinxin Zhang, Yage Zhao

**Affiliations:** 1 Hubei University of Chinese Medicine, Wuhan, Hubei, China; 2 School of Pediatrics, Henan University of Chinese Medicine, Zhengzhou, Henan, China; 3 Department of Pediatrics, The First Affiliated Hospital of Henan University of Traditional Chinese Medicine, Zhengzhou, Henan, China; 4 Macau University of Science and Technology, Macao, Macao SAR, China

**Keywords:** clinical decision support, conservative Q-learning, glucocorticoids, reinforcement learning, sepsis

## Abstract

**Background:**

The use of glucocorticoids in sepsis remains controversial due to heterogeneous treatment responses across patient populations. More individualized treatment strategies are needed to guide glucocorticoid administration.

**Methods:**

An offline reinforcement learning (RL) model was developed using the MIMIC-IV database and externally validated in the eICU-CRD. Adult patients with sepsis were included, and clinical trajectories were constructed using 24-hour time steps. Least absolute shrinkage and selection operator (LASSO) regression was applied for feature selection, and missing data were handled using multiple imputation. The problem was formulated as a Markov decision process, with glucocorticoid dosing defined as discrete actions. A conservative Q-learning (CQL) algorithm was used to learn treatment policies. Policy performance was evaluated using fitted Q-evaluation (FQE), and comparisons were made with clinician strategies. SHAP analysis was performed to interpret model decisions.

**Results:**

A total of 3,070 patients from the MIMIC-IV cohort and 372 from the eICUCRD cohort were included. The CQL policy achieved higher expected returns than clinician policy in both datasets. Higher expected returns were associated with higher survival rates. The model recommended a more selective glucocorticoid use pattern, with reduced overall usage and delayed initiation in some patients.

**Conclusion:**

The RL model identified individualized glucocorticoid treatment strategies and demonstrated improved policy performance compared with clinician practice, suggesting its potential as a clinical decision support tool.

## 1 Introduction

Sepsis is defined as life-threatening organ dysfunction caused by a dysregulated host response to infection and remains a major cause of mortality worldwide, with reported mortality rates of approximately 20–30% (Fleischmann et al., 2016; Singer et al., 2016). The use of glucocorticoids in the treatment of sepsis remains highly controversial (Teja et al., 2024; Annane et al., 2025). Glucocorticoids exert potent anti-inflammatory effects by suppressing the production of proinflammatory cytokines, but they may also impair immune responses that are essential for pathogen clearance (Rhen and Cidlowski, 2005). While some studies have suggested beneficial effects of glucocorticoid therapy in sepsis, including reduced 28-day mortality and shorter ICU length of stay, other studies have reported neutral or even adverse outcomes (Annane et al., 2002; Sprung et al., 2008; Venkatesh et al., 2018). These inconsistent findings may partly reflect the heterogeneous of sepsis, which encompasses multiple subphenotypes that may respond differently to glucocorticoid therapy (Antcliffe et al., 2019). Therefore, more individualized and precise treatment strategies are needed to guide glucocorticoid use in patients with sepsis.

Reinforcement learning (RL) is a machine learning approach that aims to optimize sequential decisionmaking by learning policies through interactions with the environment (Mnih et al., 2015; Silver et al., 2016; Van Hasselt et al., 2016; Vincent et al., 2018).Traditional statistical methods and most machine learning studies in critical care have primarily focused on outcome prediction rather than optimizing treatment strategies (Kim et al., 2025). In recent years, RL has attracted increasing attention in healthcare research and has been applied to guide clinical decision-making, including fluid resuscitation, medication dosing, and ventilatory support (Mollura et al., 2022; Lee et al., 2024; Liu et al., 2024; Zhou et al., 2025). These findings highlight the potential of RL to support optimized and individualized treatment decisions.

In this study, we developed an RL–based clinical decision support system using large critical care databases and performed external validation. The system was designed to assist glucocorticoid treatment strategies for patients with sepsis, focusing on treatment timing, dosing, and duration. This approach has the potential to provide clinicians with more precise and individualized guidance and may ultimately improve clinical outcomes.

## 2 Methods

### 2.1 Data source

This study was conducted using two publicly accessible critical care datasets: the Medical Information Mart for Intensive Care IV (MIMIC-IV) and the eICU Collaborative Research Database (eICU-CRD) (Pollard et al., 2019; Johnson et al., 2024). The first author obtained authorised access to both databases after completing the required training (Record ID: 74618451). Given that all data were de-identified, informed consent was not required.

### 2.2 Study population

**Figure 1:**
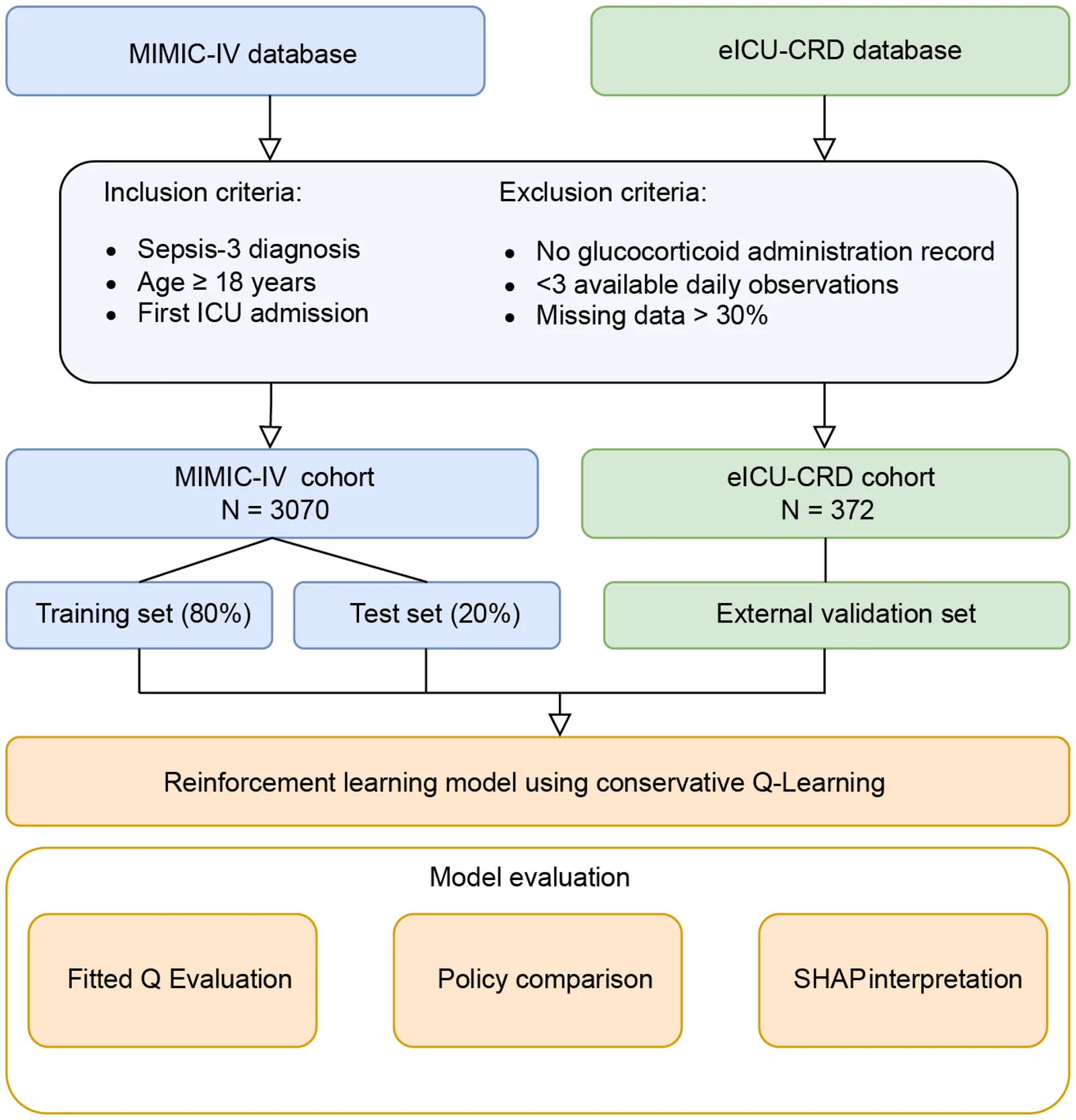
Study design flowchart. Overview of patient selection, model development using conservative Q-learning, and model evaluation.

The patient inclusion criteria were as follows (1): patients diagnosed with sepsis according to the Sepsis-3 criteria (2); patients aged ≥18 years; and (3) patients during their first ICU admission. The exclusion criteria were as follows (1): patients without records of glucocorticoid administration (2); patients with fewer than three available daily observations within the early ICU trajectory window; and (3) patients with missing values exceeding 30% among the study variables. Patients with fewer than three available daily observations were excluded because such short trajectories provide insufficient sequential information for constructing state transitions in the RL framework. For each eligible patient, the RL trajectory was constructed from ICU admission and followed until the earliest of the following events: completion of the first seven ICU days, ICU discharge, or death. The first seven ICU days corresponded to day intervals 1–7, and each interval was discretized into a 24-hour decision step. The overall study workflow is illustrated in **Figure 1**.

### 2.3 Data extraction

Structured Query Language (SQL) was used to extract clinical data from the databases. Extracted variables were categorized into demographic characteristics, treatment-related variables, vital signs, laboratory measurements, severity scores, and clinical outcomes. A complete list of extracted variables is provided in **Supplementary Table 1**. The included systemic glucocorticoids were hydrocortisone, prednisone, and methylprednisolone. All glucocorticoid doses were converted to hydrocortisone-equivalent doses before action construction, using conversion factors of 1 for hydrocortisone, 4 for prednisone, and 5 for methylprednisolone, consistent with standard glucocorticoid equivalency tables (Nicolaides et al., 2018).

### 2.4 Data preprocessing

Least absolute shrinkage and selection operator (LASSO) regression was applied to identify relevant predictors from 29 clinically relevant candidate variables selected from prior literature and clinical plausibility (Komorowski et al., 2018). In-hospital death was used as the response variable in the LASSO logistic regression. Continuous variables were min–max normalized, categorical variables were treated as factors, and the tuning parameter *λ* was selected via 10-fold cross-validation by minimising the binomial deviance. Variables with non-zero coefficients at the selected *λ* were retained for subsequent modelling. In total, 17 variables were ultimately selected to define the state representation of the RL model. Each state represented the observed clinical condition within a 24-hour interval. Because vital signs, laboratory values, severity scores, fluid administration, and treatment variables were aggregated over each 24-hour window, the state representation implicitly incorporated short-term historical information from the preceding interval. However, we did not explicitly stack multiple previous time steps or use a recurrent encoder in the primary model.

Missing data were first handled by forward filling, and any remaining missing values were subsequently imputed using multiple imputation by chained equations (MICE) (White et al., 2011). The distributions before and after imputation were evaluated using kernel density plots for each variable as a visual check of imputation performance (**Supplementary Figure 1**).

To explore the structure of the state space, t-distributed stochastic neighbour embedding (t-SNE) was applied to visualise the high-dimensional state representations. Each point in the t-SNE plot represents one patient-time state, defined as the selected clinical variables within a 24-hour ICU interval. In the reinforcement learning dataset, a patient trajectory consists of a sequence of such patient-time states across consecutive intervals (Van der Maaten and Hinton, 2008).

### 2.5 Model development

A Markov Decision Process (MDP) framework was adopted to model the sequential decision-making problem, represented by the tuple (S,A,R,γ) (Sutton and Barto, 2018). The state space S is multi-dimensional and consists of 17 clinical features. The action space A is discrete and corresponds to the dosage of hydrocortisoneequivalent glucocorticoid: doses range from 0 to 400 mg with a step size of 50 mg and an additional category for doses greater than 400 mg. The reward function R is anchored by the terminal survival outcome, with -1 assigned for death and +1 for survival. To address reward sparsity, we additionally evaluated a shaped reward that retained the terminal reward and added bounded intermediate terms for SOFA improvement and reduction in hyperglycemia above 180 mg/dL. The discount factor *γ* is set to 0.99, which indicates that the agent places high importance on long-term outcomes. The shaped reward was defined as Equation 1

(1) $$r_{t}=r_{terminal}+0.1\times \text{clip}((SOFA_{t}-SOFA_{t+1})/4,-1,1)+0.05\times \text{clip}((E_{t}-E_{t+1})/100,-1,1),$$

where *E_t_* = max(*glucose_t_*−180,0). Thus, glucose values at or below 180 mg/dL were not penalized. The SOFA and glucose intermediate components were bounded within ±0.10 and ±0.05, respectively, so that intermediate rewards did not override the terminal mortality outcome. The objective is to learn an optimal policy *π*(*a*|*s*) from an offline dataset that predicts the most appropriate dosage for given patient states. In this offline reinforcement learning setting, distributional shift is a central challenge: the learned policy may select actions not well represented in the training data, leading to overestimation of values (Levine et al., 2020).

Conservative Q-learning (CQL) augments the Bellman error with a regularization term that penalizes out-of-distribution actions while favoring actions observed in the dataset, yielding a conservative estimate of the value function and improving policy reliability (Kumar et al., 2020).. The process of the above algorithm is shown in **Algorithm 1**. The conservative Q-function tradeoff factor *α* was set to 0.5, the batch size B to 256, the discount factor *γ* to 0.99, and the learning rate *τ* to 3 × 10^−5^. The Q-function is represented by a neural network consisting of an input layer, two fully-connected hidden layers each with 256 units and tanh activation, and an output layer. Optimization was performed using the Adam optimizer, with the decay rates for the first and second moment estimates set to 0.9 and 0.99, respectively.

Algorithm 1

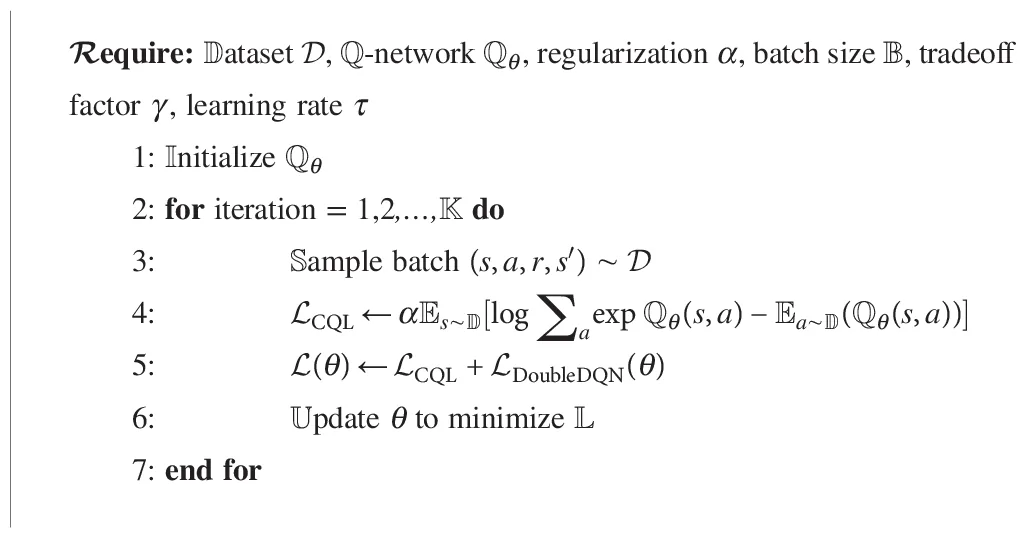

The CQL model was developed using MIMIC-IV data only. Feature selection, model training, and hyperparameter tuning were performed within the MIMIC-IV cohort. After training, the learned policy was fixed and applied directly to the previously unseen eICU-CRD cohort for external validation. The eICU-CRD data were not used for feature selection, model training, or hyperparameter tuning.

### 2.6 Policy evaluation

Policy performance was evaluated using Fitted Q-Evaluation (FQE), an off-policy evaluation (OPE) method (Le et al., 2019). FQE iteratively estimates the Q-function for the target policy by minimizing the Bellman error on the offline validation dataset (Equation 2):

(2) $$Q_{k+1}^{\pi }\leftarrow \text{arg }\underset{f}{\min}E_{(s,a,r,s^{\prime })∼D}[{\left(r+\gamma Q_{k}^{\pi }(s^{\prime },\pi (s^{\prime }))-f(s,a)\right)}^{2}]$$

This process converges to an estimate of *Q^π^*, providing a robust and sample-efficient way to assess policy performance. Database was divided using an 8:2 split for training and validation sets to compare the performance of the CQL policy and the clinician policy. To assess the generalization capability of the model, the eICU-CRD database was employed as an external validation set for performance comparison.

To elucidate the clinical significance of the Q-values obtained from policy evaluation, we analyzed the relationship between the Q-values and mortality rates. Specifically, we employed an offline state–action–reward–state–action (SARSA) algorithm incorporating the Bellman optimality equation (Sutton and Barto, 2018). The SARSA algorithm was trained using randomly sampled sequences of consecutive patient transitions. The action-value function update in SARSA is given by Equation 3:

(3) $$Q^{\pi }(s,a)\leftarrow Q^{\pi }(s,a)+\alpha [R+\gamma Q^{\pi }(s^{\prime },a^{\prime })-Q^{\pi }(s,a)].$$

**Figure 2:**
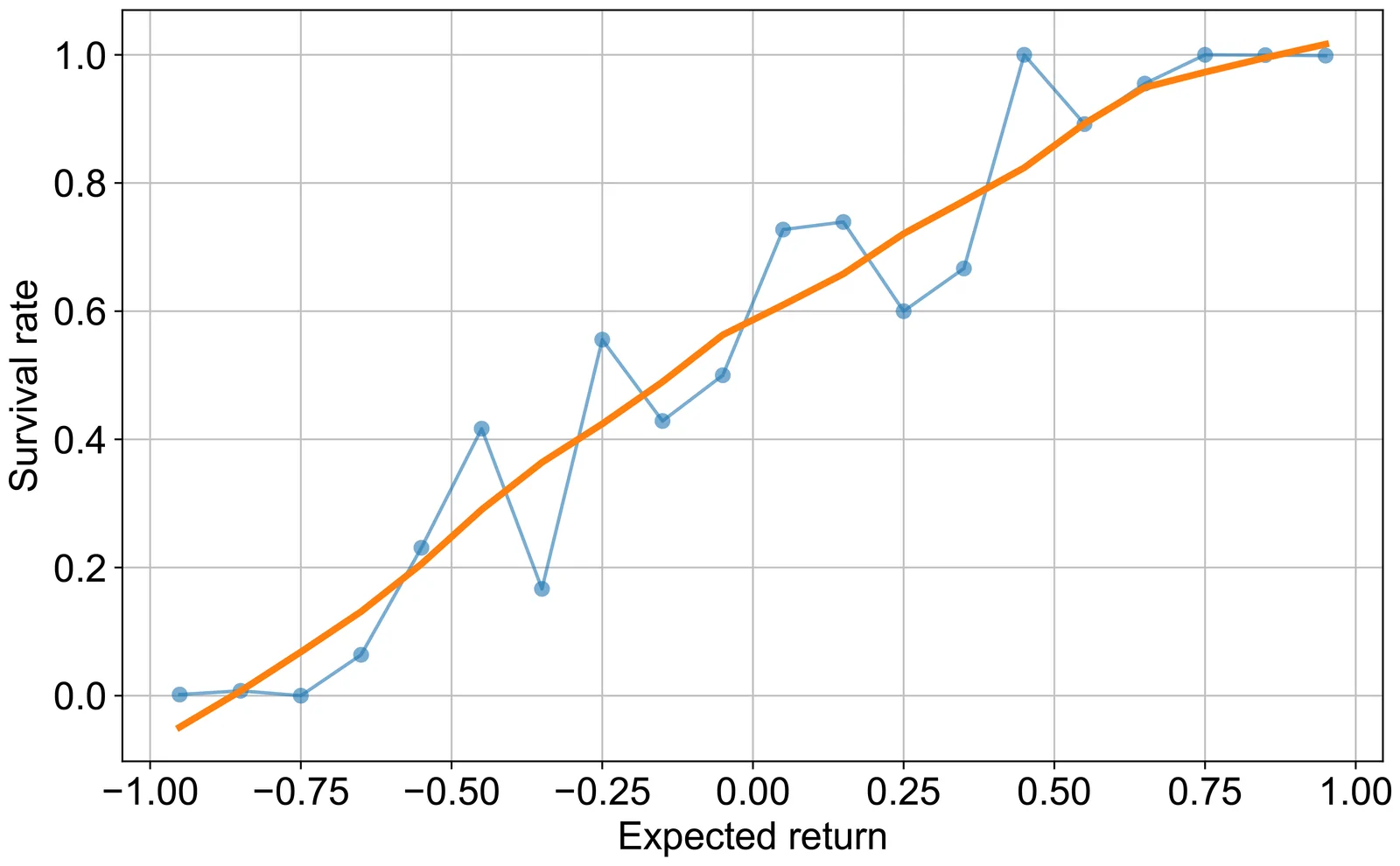
Observed survival rate vs. expected return curve of MIMIC-IV. The x axis represents expected return and the y axis represents the observed survival rate.

Expected returns were estimated for each patient using Q-values derived from the SARSA algorithm. Patients were stratified into 20 bins based on expected return, and the observed average survival rate within each bin was calculated. Therefore, the survival rate shown in **Figure 2** represents the observed survival rate within each expected-return stratum rather than a predicted survival probability. Higher expected returns were associated with increased survival rates. The SARSA analysis used a patient-level train/test split of 8:2, discount factor *γ* = 0.99, learning rate 1 × 10^−4^, Adam optimizer, batch size 256, a Q-network with two 256 unit hidden layers and ReLU activation.

### 2.7 Policy comparison and interpretability

Action selection under the CQL and clinician policies was compared using heatmaps of glucocorticoid administration frequency and timing, along with patient trajectories to qualitatively assess dynamic treatment patterns over the ICU stay.

To interpret the decision-making process of the CQL policy and clinician policy, We employed SHAP (SHapley Additive exPlanations) (Lundberg and Lee, 2017). SHAP quantify the importance of each state feature by computing its SHapley value in determining policy’s recommended glucocorticoid dosage, thereby identifying the key drivers of the two policies.

### 2.8 Statistical analysis

Continuous variables were summarized as medians with interquartile ranges and compared between cohorts using the two-sided Mann–Whitney U test. Categorical variables were summarized as counts and percentages and compared using Pearson’s chi-square test without continuity correction. Glucocorticoid dose distributions were summarized descriptively using counts, percentages, and heatmaps showing dose categories across time steps. These dose distribution analyses were interpreted as descriptive comparisons of policy behavior rather than causal hypothesis tests at the patient level.

## 3 Results

### 3.1 Baseline characteristics of patients

A total of 3070 patients with sepsis were included from MIMIC-IV, of whom 1,185 (38.6%) expired, and 372 patients with sepsis were included from eICU-CRD, of whom 120 (32.3%) expired.

A summary of baseline characteristics is provided in **Table 1**. Significant differences were observed in several clinical parameters. Patients in the eICU-CRD cohort had higher median SOFA scores (9.0 vs. 6.0, *P <* 0.001) and higher RASS scores (-1.0 vs. -3.0, *P <* 0.001). Additionally, heart rate, PT, PTT, and BUN levels were significantly higher in the eICU-CRD cohort (all *P <* 0.001). In contrast, base excess and SBP were lower in the eICU-CRD cohort (all *P <* 0.001). Respiratory rate was comparable between the two groups, with no significant difference observed. (*P >* 0.05).

**Table 1:** Baseline characteristics of patients.

Dataset	Variable	MIMIC-IV	eICU-CRD	*P*-value
Dataset characteristics	Patients	3070	372	
	Gender			0.346
	Female(%)	1474 (48.0%)	169 (45.4%)	
	Male(%)	1596 (52.0%)	203 (54.6%)	
	Mortality (%)	1185 (38.6%)	120 (32.3%)	0.017
Patient characteristics at the start of trajectories	Age	66.0 (56.0, 76.0)	63.0 (52.0, 72.0)	*<* 0.001
	Weight	79.8 (66.9, 95.9)	83.0 (66.5, 100.3)	0.084
	SOFA	6.0 (4.0, 9.0)	9.0 (5.0, 12.0)	*<* 0.001
	RASS	-3.0 (-4.0, -1.0)	-1.0 (-2.0, 0.0)	*<* 0.001
	Heart rate	89.6 (77.5, 102.0)	96.0 (83.2, 107.8)	*<* 0.001
	Respiratory rate	20.8 (17.9, 24.2)	20.6 (17.3, 24.1)	0.319
	PT	14.6 (12.9, 18.2)	17.9 (14.6, 23.6)	*<* 0.001
	PTT	33.3 (28.1, 42.5)	39.7 (32.5, 54.0)	*<* 0.001
	BUN	27.0 (17.0, 43.0)	34.4 (21.6, 50.0)	*<* 0.001
	Hemoglobin	10.0 (8.7, 11.6)	9.7 (8.3, 11.3)	*<* 0.01
	SpO_2_	96.6 (94.9, 98.1)	97.0 (95.2, 98.5)	*<* 0.01
	Base excess	-1.0 (-5.0, 1.5)	-2.7 (-7.1, 0.8)	*<* 0.001
	SBP	112.0 (104.1, 123.0)	109.5 (99.2, 121.1)	*<* 0.001
	Glucose	145.3 (117.3, 184.3)	144.0 (117.9, 184.2)	0.647

SOFA, sequential organ failure assessment; RASS, Richmond Agitation-Sedation Scale; PT, prothrombin time; PTT, partial thromboplastin time; BUN, blood urea nitrogen; SpO_2_, peripheral oxygen saturation; SBP, systolic blood pressure. Continuous variables are presented as median (interquartile range).

### 3.2 Feature selection

Feature selection was performed using LASSO regression shown in **Supplementary Figure 2**. The 17 selected predictors of death included gender, age, weight, temperature, Sequential Organ Failure Assessment (SOFA) score, Richmond Agitation Sedation Scale (RASS) score, heart rate, respiratory rate, prothrombin time (PT), partial thromboplastin time (PTT), blood urea nitrogen (BUN), hemoglobin, peripheral oxygen saturation (SpO_2_), base excess, systolic blood pressure (SBP), total intravenous fluid volume, and glucose.

### 3.3 State space comparison

**Figure 3:**
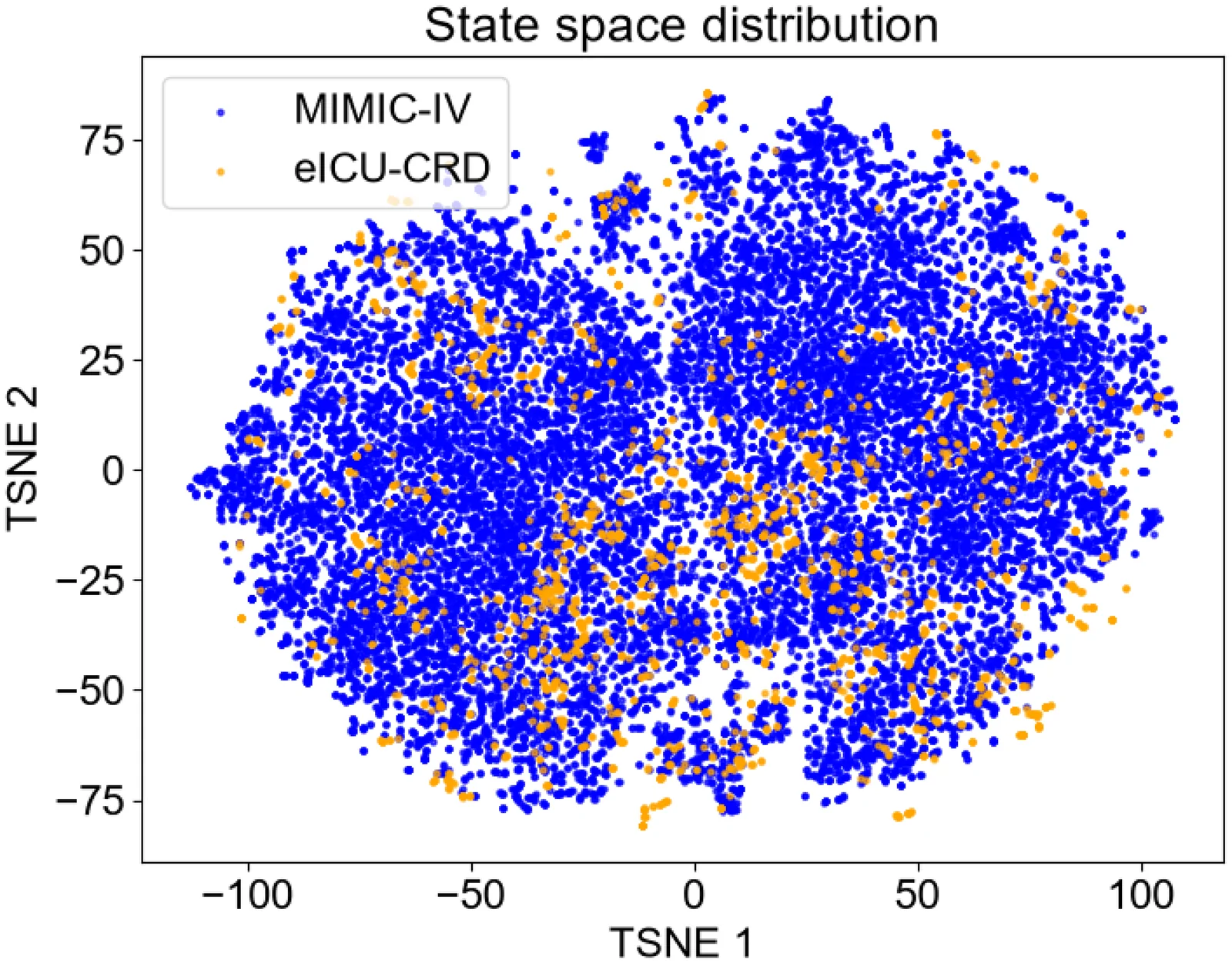
State space distribution.Two-dimensional t-SNE visualization of patient states derived from the MIMIC-IV and eICU-CRD databases. Each point represents a patient-time state, with colors indicating different data sources.

The t-SNE visualization of the state space demonstrated a largely overlapping distribution between the MIMIC-IV and eICU-CRD (**Figure 3**). Data points from both databases were widely intermixed without clear separation, suggesting a comparable distribution of patient states across the two datasets. Although the MIMIC-IV cohort exhibited a higher density due to the larger sample size, the overall structural patterns were consistent.

### 3.4 Policy evaluation

**Table 2** summarizes the policy evaluation results, where CQL policy has higher mean expected return in both MIMIC-IV (1.94) and eICU-CRD datasets (1.82). Furthermore, in both the MIMIC-IV and eICU-CRD datasets, the CQL policy’s 95% confidence interval lower bound lies above the clinician policy’s 95% confidence interval upper bound.

**Table 2:** Policy evaluation results with fitted-Q evaluation.

Dataset	CQL policy		Clinician policy	
	Mean	95% CI	Mean	95% CI
MIMIC-IV	1.94	(1.91, 1.96)	1.84	(1.82, 1.87)
eICU-CRD	1.82	(1.78, 1.85)	1.71	(1.67, 1.74)

Based on the relationship between the observed survival rate and the expected return illustrated in **Figure 2**, the higher Q-value represents the higher observed survival rate.

To examine clinical applicability across disease severity, we performed subgroup analyses stratified by baseline SOFA score. The CQL policy showed consistently higher FQE-estimated value than the clinician policy across SOFA *<* = 6, SOFA 7–10, and SOFA *>* = 11 strata in both MIMIC-IV and eICU-CRD. The CQL-minus-clinician value differences were +0.093, +0.099, and +0.092 in MIMIC-IV, and +0.111, +0.117, and +0.107 in eICU-CRD, respectively. Additional exploratory subgroup analyses by age, gender, glucose, systolic blood pressure, blood urea nitrogen, RASS, and initial glucocorticoid dose showed generally positive CQL advantages (**Supplementary Figure 4**).

### 3.5 Policy analysis and comparison

**Figure 4:**
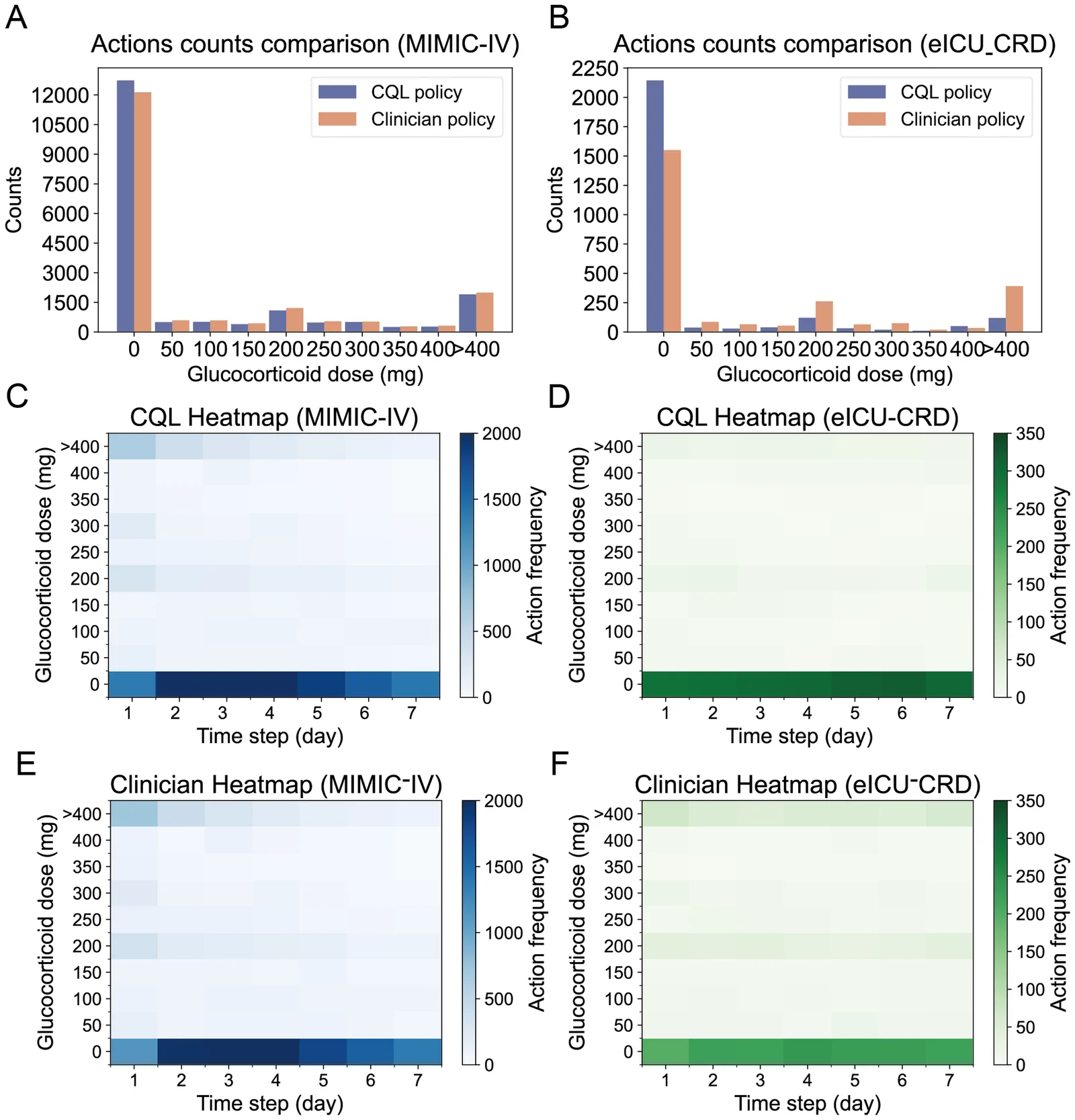
Comparison of glucocorticoid dosing strategies between the CQL policy and clinician policy across two databases. **(A, B)** Distribution of dose categories in the MIMIC-IV and eICU-CRD cohorts. **(C, D)** Heatmaps of action distributions across time steps under the CQL policy. **(E, F)** Heatmaps of action distributions under the clinician policy.

The overall usage of glucocorticoid was analyzed in the MIMIC-IV and eICU-CRD databases. In **Figures 4A, B**, compared with the clinician policy, an increase in the number of absence of use of glucocorticoid and a decrease in the number for each of the remaining dose selection were observed for the CQL policy in both databases. Furthermore, the dose selection at each time step was further demonstrated by the heatmap presented in **Figures 4C–F**.

**Figure 5:**
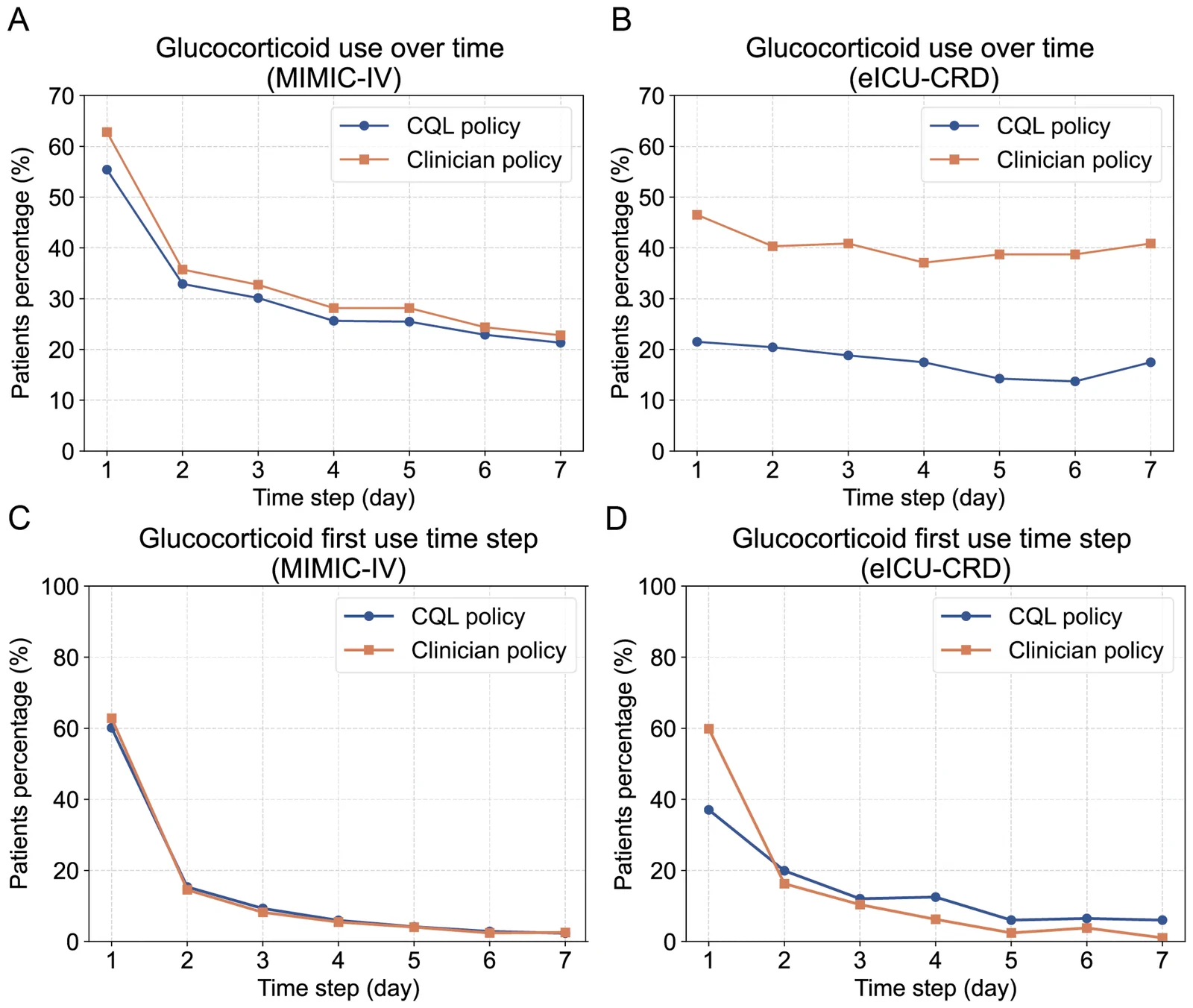
Comparison of glucocorticoid use and timing of first administration between the CQL policy and clinician policy in the MIMIC-IV and eICU-CRD datasets. **(A, B)** Percentage of patients receiving glucocorticoids at each time step in the MIMIC-IV and eICU-CRD cohorts. **(C, D)** Distribution of the first glucocorticoid administration time step.

The proportion of patients receiving glucocorticoids at different time steps was analyzed (**Figures 5A, B**). In the MIMIC-IV database, a consistent pattern was observed between the CQL policy and the clinician policy, with the highest proportion of patients receiving medication on the first day of ICU admission, reaching 55.41% and 62.80%, respectively, after which the proportions gradually decreased. In the eICUCRD database, the highest proportion of patients receiving medication was also observed on the first day under both policies, reaching 21.51% and 46.51%, respectively, after which the proportion decreased slightly under the CQL policy but remained relatively stable under the clinician policy. Across both databases, the proportion of patients receiving medication under the CQL policy was lower than that under the clinician policy at each time step.

The timing of first glucocorticoid administration was also analyzed (**Figures 5C, D**). In both databases, a declining trend was observed under both the CQL policy and the clinician policy. In the MIMIC-IV database, a high degree of consistency was observed between the two policies. In contrast, in the eICU-CRD database, the proportion of patients receiving their first medication on day 1 was lower under the CQL policy compared to the clinician policy (37.04% vs. 59.86%), while a slight increase was observed in the proportion of patients receiving their first medication between days 2 and 7.

### 3.6 SHAP analysis

SHAP analysis was performed to interpret the decision-making patterns of the CQL policy and the clinician policy (**Supplementary Figure 3**). Overall, both policies identified similar key variables, including gender, heart rate, SBP, and respiratory rate, suggesting that fundamental physiological indicators were consistently influential in treatment decisions. We interpreted SHAP as an explanation of the learned policy rather than evidence of causal treatment effects. SOFA score reflected organ dysfunction burden, blood pressure and heart rate reflected hemodynamic instability, respiratory variables reflected respiratory status, glucose reflected a steroid-related metabolic safety signal, and prior glucocorticoid exposure reflected treatment history.

## 4 Discussion

In this study, an offline RL framework was developed and externally validated using the CQL algorithm to explore the optimization of glucocorticoid administration in patients with sepsis. Data from 3,070 patients in the MIMIC-IV database were utilized for model training and testing, while data from 372 patients in the eICU-CRD database were employed for independent external validation. Higher expected returns were found to be achieved by the CQL-derived policy in both cohorts. A positive correlation between the model’s expected return and the survival rate was observed, suggesting that the treatment trajectories identified by the model are associated with potential survival benefits. These findings provided a data-driven alternative to the currently controversial glucocorticoid administration protocols in septic patients.

Several previous studies have applied RL to sequential treatment decisions in sepsis. Early work modeled treatment actions as combinations of intravenous fluid and vasopressor doses and reported higher estimated values for RL-recommended strategies than for observed clinician actions (Komorowski et al., 2018). Subsequent studies refined this framework in several directions. One study combined deep and kernel-based reinforcement learning to personalize fluid and vasopressor treatment according to patient history (Peng et al., 2018). Another study incorporated cardiovascular physiological modeling and uncertainty-aware decision support for sepsis treatment control (Nanayakkara et al., 2022). Mollura et al. examined the effect of feature-space dimensionality reduction on RL model performance for fluid and vasopressor interventions in sepsis (Mollura et al., 2022). More recent studies have expanded the scope of sepsis RL. One study extracted treatment policies directly from hospital treatment records using deep reinforcement learning (Choi et al., 2024). Another study incorporated medical knowledge and disease severity to improve individualized recommendations for heterogeneous sepsis patients (Feng et al., 2025). The OVISS study used reinforcement learning to estimate an optimal rule for vasopressin initiation in septic shock and evaluated this rule across multiple hospital datasets (Kalimouttou et al., 2025). These studies indicate that RL-based treatment policies are strongly influenced by state representation, reward specification, action support, and off-policy evaluation.

Compared with standard Q-learning or deep Q-network approaches, CQL is specifically designed for offline reinforcement learning, where policies are learned from fixed historical datasets without further interaction with the environment. In this setting, distributional shift between the learned policy and the behavior policy can lead to overestimation of rarely observed or out-of-distribution actions. CQL mitigates this issue by learning a conservative Q-function that penalizes unsupported actions and provides a lower-bound estimate of policy value, which is particularly relevant for medication dosing decisions in retrospective ICU data (Gottesman et al., 2019; Kumar et al., 2020; Levine et al., 2020).

The generalizability of RL models in the ICU is often constrained by the inherent heterogeneity of patient populations across different institutions (Gottesman et al., 2019). In this analysis, significantly higher baseline severity was exhibited by patients in the eICU-CRD cohort, as evidenced by higher median SOFA scores and more pronounced physiological derangements in heart rate and coagulation parameters (PT, PTT) compared to the MIMIC-IV cohort. However, a largely overlapping distribution between the two databases was demonstrated by the t-SNE visualization of the state space, with data points found to be widely intermixed. This similarity supports the empirical feasibility of external evaluation, but t-SNE overlap alone cannot establish full transportability. We therefore interpreted the external validation and SOFA-stratified subgroup analysis as robustness checks rather than definitive proof that dataset shift is absent.

Differences in medication actions were revealed through a comparison between the CQL policy and clinician behavior across both cohorts. The CQL model consistently favored a more selective approach, characterized by a lower frequency of intervention at each time step compared to actual clinical practice. This finding is consistent with prior observations (Lee et al., 2024; Desman et al., 2025). While a peak in treatment initiation on the first day of ICU admission was exhibited by both policies, a more cautious distribution was demonstrated by the RL model, with a preference for zero-dosage and a restricted use of high-dose regimens. The timing of the first administration further highlights the differences between the two policies. Notably, a strategy of delayed initiation was explored by the RL agent in the eICU-CRD cohort, where treatment was deferred for a subset of patients during the initial 24 hours. This suggests that a more stringent requirement for definitive physiological signals may be captured by the model before committing to steroid therapy. These findings indicate that the RL clinical decision support tool may assist in identifying the optimal therapeutic window, potentially minimizing unnecessary steroid exposure in heterogeneous clinical settings.

The physiological determinants underlying the model’s decisions were further characterized through SHAP analysis. Gender, heart rate, SBP, and respiratory rate were identified by SHAP as the most influential drivers of the RL policy, showing a feature importance ranking that is highly consistent with the observed clinician behavior. This alignment suggests that the learned policy used clinically recognizable physiological information, but SHAP does not establish causal mechanisms between these variables, glucocorticoid administration, and outcomes. This pattern is consistent with prior machine learning studies in sepsis, where vital signs and basic physiological parameters have been repeatedly identified as dominant predictors of clinical outcomes (Hu et al., 2022; Zhou et al., 2024; Athukorala and Ilmini, 2026).

Several limitations of this study should be acknowledged. First, the use of historical clinical data for model development may lead to biases caused by recording errors and missing values. The observed state vector is only a pragmatic approximation of the latent clinical state, and the strict Markov assumption is unlikely to be fully satisfied in ICU sepsis trajectories. Unmeasured infection source control, antimicrobial decisions, clinician judgment, earlier treatment history, and latent disease trajectory may not be fully captured. Second, offline RL cannot fully substitute for real-time validation, and the actual clinical impact of the recommendations requires testing in prospective interventional trials. Although CQL reduces overestimation of poorly supported actions, it cannot remove treatment-selection bias, residual confounding, or measurement error in observational data. The learned policy should therefore be interpreted as hypothesis generating decision support rather than a causal treatment rule. Third, geographical and institutional variations in clinical practice patterns may still exist despite the use of multicenter databases, which could affect the generalizability of the model to different healthcare systems. Fourth, the 24-hour decision interval improves data completeness and comparability but may miss rapid hemodynamic deterioration, vasopressor escalation, and transient adverse events. Fifth, the reward design and outcome assessment did not fully capture all steroid-related safety endpoints, including secondary infection, ICU length of stay, and other adverse effects.

## 5 Conclusions

In this study, a clinical decision support tool was developed and validated using an offline RL framework to identify individualized glucocorticoid administration patterns in sepsis. The model achieved higher expected rewards in both datasets. Policy analysis indicated that the RL policy favored a more selective and conservative strategy compared to clinician behavior. These findings suggest that this data-driven approach could provide valuable support for clinicians managing complex septic patients.

## Data Availability

Publicly available datasets were analyzed in this study. This data can be found here: The datasets for this study can be found in the https://physionet.org/content/mimiciv/3.1/ and https://physionet.org/content/eicu-crd/2.0/.

## References

[B1] Annane D. Briegel J. Seymour C. W. (2025). Corticosteroids in sepsis: certainties and shadows. Intensive Care Med. 51, 1890–1893. doi: 10.1007/s00134-025-08044-3 40699324

[B2] Annane D. Sebille´ V. Charpentier C. Bollaert P. E. Franc¸ois B. Korach J. M. . (2002). Effect of treatment with low doses of hydrocortisone and fludrocortisone on mortality in patients with septic shock. JAMA 288, 862–871. doi: 10.1001/jama.288.7.862 12186604

[B3] Antcliffe D. B. Burnham K. L. Al-Beidh F. Santhakumaran S. Brett S. J. Hinds C. J. . (2019). Transcriptomic signatures in sepsis and a differential response to steroids. from the vanish randomized trial. Am. J. Respir. Crit. Care Med. 199, 980–986. doi: 10.1164/rccm.201807-1419oc 30365341 PMC6467319

[B4] Athukorala V. Ilmini W. (2026). Explainable ai for critical care: a systematic review of interpretable models for sepsis and icu mortality prediction. BMC Med. Inf. Decis. Making 26, 164. doi: 10.1186/s12911-026-03344-0 41634648 PMC12955129

[B5] Choi Y. Oh S. Huh J. W. Joo H. T. Lee H. You W. . (2024). Deep reinforcement learning extracts the optimal sepsis treatment policy from treatment records. Commun. Med. 4, 245. doi: 10.1038/s43856-024-00665-x 39578542 PMC11584651

[B6] Desman J. M. Hong Z. W. Sabounchi M. Sawant A. S. Gill J. Costa A. C. . (2025). A distributional reinforcement learning model for optimal glucose control after cardiac surgery. NPJ Digital Med. 8, 313. doi: 10.1038/s41746-025-01709-9 40425725 PMC12116759

[B7] Feng X. Zhu S. Fang L. Zhu H. Cai G. Shen Y. . (2025). Tailored to fit sepsis individuals: Medical knowledge aware reinforcement learning model offers optimized therapeutic strategies. IEEE J. Biomed. Health Inf. 30, 5692–5705. doi: 10.1109/jbhi.2025.3647877 41442288

[B8] Fleischmann C. Scherag A. Adhikari N. K. Hartog C. S. Tsaganos T. Schlattmann P. . (2016). Assessment of global incidence and mortality of hospital-treated sepsis. current estimates and limitations. Am. J. Respir. Crit. Care Med. 193, 259–272. doi: 10.1164/rccm.201504-0781oc 26414292

[B9] Gottesman O. Johansson F. Komorowski M. Faisal A. Sontag D. Doshi-Velez F. . (2019). Guidelines for reinforcement learning in healthcare. Nat. Med. 25, 16–18. doi: 10.1038/s41591-018-0310-5 30617332

[B10] Hu C. Li L. Huang W. Wu T. Xu Q. Liu J. . (2022). Interpretable machine learning for early prediction of prognosis in sepsis: a discovery and validation study. Infect. Dis. Ther. 11, 1117–1132. doi: 10.1007/s40121-022-00628-6 35399146 PMC9124279

[B11] Johnson A. Bulgarelli L. Pollard T. Gow B. Moody B. Horng S. . (2024). MIMIC-IV. PhysioNet. doi: 10.13026/kpb9-mt58

[B12] Kalimouttou A. Kennedy J. N. Feng J. Singh H. Saria S. Angus D. C. . (2025). Optimal vasopressin initiation in septic shock: the oviss reinforcement learning study. JAMA 333, 1688–1698. doi: 10.1001/jama.2025.3046 40098600 PMC11920879

[B13] Kim Y. Kim M. Kim Y. Choi M. (2025). Using nursing data for machine learning-based prediction modeling in intensive care units: a scoping review. Int. J. Nurs. Stud. 169, 105133. doi: 10.1016/j.ijnurstu.2025.105133 40544524

[B14] Komorowski M. Celi L. A. Badawi O. Gordon A. C. Faisal A. A. (2018). The artificial intelligence clinician learns optimal treatment strategies for sepsis in intensive care. Nat. Med. 24, 1716–1720. doi: 10.1038/s41591-018-0213-5 30349085

[B15] Kumar A. Zhou A. Tucker G. Levine S. (2020). Conservative q-learning for offline reinforcement learning. Adv. Neural Inf. Process. Syst. 33, 1179–1191.

[B16] Le H. Voloshin C. Jiang N. (2019). “ Batch policy learning under constraints”, in Proceedings of the 36th International Conference on Machine Learning, Proceedings of Machine Learning Research, vol. 97, ( PMLR), 3703–3712.

[B17] Lee H. Y. Chung S. Hyeon D. Yang H. L. Lee H. C. Ryu H. G. . (2024). Reinforcement learning model for optimizing dexmedetomidine dosing to prevent delirium in critically ill patients. NPJ Digital Med. 7, 325. doi: 10.1038/s41746-024-01335-x 39557970 PMC11574043

[B18] Levine S. Kumar A. Tucker G. Fu J. (2020). Offline reinforcement learning: Tutorial, review, and perspectives on open problems. arXiv Preprint arXiv:2005.01643.

[B19] Liu S. Xu Q. Xu Z. Liu Z. Sun X. Xie G. . (2024). Reinforcement learning to optimize ventilator settings for patients on invasive mechanical ventilation: retrospective study. J. Med. Internet Res. 26, e44494. doi: 10.2196/44494 39219230 PMC11525081

[B20] Lundberg S. M. Lee S. I. (2017). A unified approach to interpreting model predictions. Adv. Neural Inf. Process. Syst. 30.

[B21] Mnih V. Kavukcuoglu K. Silver D. Rusu A. A. Veness J. Bellemare M. G. . (2015). Human-level control through deep reinforcement learning. Nature 518, 529–533. doi: 10.1038/nature14236 25719670

[B22] Mollura M. Drudi C. Lehman L. W. Barbieri R. (2022). “ A reinforcement learning application for optimal fluid and vasopressor interventions in septic icu patients”, in: 2022 44th Annual International Conference of the IEEE Engineering in Medicine & Biology Society (EMBC) (Glasgow: IEEE), 321–324. doi: 10.1109/EMBC48229.2022.9871055 36086153

[B23] Nanayakkara T. Clermont G. Langmead C. J. Swigon D. (2022). Unifying cardiovascular modelling with deep reinforcement learning for uncertainty aware control of sepsis treatment. PloS Digital Health 1, e0000012. doi: 10.1371/journal.pdig.0000012 36812511 PMC9931225

[B24] Nicolaides N. C. Pavlaki A. N. Maria Alexandra M. A. Chrousos G. P. (2018). “ Glucocorticoid therapy and adrenal suppression,” in Endotext ( MDText.com, Inc, South Dartmouth (MA).

[B25] Peng X. Ding Y. Wihl D. Gottesman O. Komorowski M. Lehman L. . (2018). “ Improving sepsis treatment strategies by combining deep and kernel-based reinforcement learning”, in AMIA Annual Symposium Proceedings, vol. 2018, ( American Medical Informatics Association) 887. PMC6371300 30815131

[B26] Pollard T. Johnson A. Raffa J. Celi L. A. Badawi O. Mark R. (2019). eICU collaborative research database. PhysioNet. doi: 10.13026/C2WM1R PMC6132188 30204154

[B27] Rhen T. Cidlowski J. A. (2005). Antiinflammatory action of glucocorticoids—new mechanisms for old drugs. N. Engl. J. Med. 353, 1711–1723. doi: 10.1056/nejmra050541 16236742

[B28] Silver D. Huang A. Maddison C. J. Guez A. Sifre L. Van Den Driessche G. . (2016). Mastering the game of go with deep neural networks and tree search. Nature 529, 484–489. doi: 10.1038/nature16961 26819042

[B29] Singer M. Deutschman C. S. Seymour C. W. Shankar-Hari M. Annane D. Bauer M. . (2016). The third international consensus definitions for sepsis and septic shock (sepsis-3). JAMA 315, 801–810. doi: 10.1001/jama.2016.0287 26903338 PMC4968574

[B30] Sprung C. L. Annane D. Keh D. Moreno R. Singer M. Freivogel K. . (2008). Hydrocortisone therapy for patients with septic shock. N. Engl. J. Med. 358, 111–124. doi: 10.1056/nejmoa071366 18184957

[B31] Sutton R. S. Barto A. G. (2018). Reinforcement Learning: An Introduction, 2nd Edn. (Cambridge, MA: MIT press).

[B32] Teja B. Berube M. Pereira T. V. Law A. C. Schanock C. Pang B. . (2024). Effectiveness of fludrocortisone plus hydrocortisone versus hydrocortisone alone in septic shock: a systematic review and network metaanalysis of randomized controlled trials. Am. J. Respir. Crit. Care Med. 209, 1219–1228. doi: 10.1164/rccm.202310-1785oc 38271488 PMC11146553

[B33] Van der Maaten L. Hinton G. (2008). Visualizing data using t-sne. J. Mach. Learn. Res. 9, 2579–2605.

[B34] Van Hasselt H. Guez A. Silver D. (2016). “ Deep reinforcement learning with double q-learning,” in Proceedings of the Thirtieth AAAI Conference on Artificial Intelligence (Phoenix, AZ: AAAI Press), 2094–2100. doi: 10.1609/aaai.v30i1.10295

[B35] Venkatesh B. Finfer S. Cohen J. Rajbhandari D. Arabi Y. Bellomo R. . (2018). Adjunctive glucocorticoid therapy in patients with septic shock. N. Engl. J. Med. 378, 797–808. doi: 10.1056/NEJMoa170583 29347874

[B36] Vincent F. L. Peter H. Riashat I. Marc G. B. Joelle P. (2018). An introduction to deep reinforcement learning. Found. Trends Mach. Learn. 11, 219–354. doi: 10.1561/2200000071

[B37] White I. R. Royston P. Wood A. M. (2011). Multiple imputation using chained equations: issues and guidance for practice. Stat. Med. 30, 377–399. doi: 10.1002/sim.4067 21225900

[B38] Zhou S. Lu Z. Liu Y. Wang M. Zhou W. Cui X. . (2024). Interpretable machine learning model for early prediction of 28-day mortality in icu patients with sepsis-induced coagulopathy: development and validation. Eur. J. Med. Res. 29, 14. doi: 10.1186/s40001-023-01593-7 38172962 PMC10763177

[B39] Zhou Y. Luo R. Blais J. E. Tan K. C. Lui D. T. W. Yiu K. H. . (2025). Optimizing long term disease prevention with reinforcement learning: a framework for precision lipid control. NPJ Digital Med. 8, 553. doi: 10.1038/s41746-025-01951-1 40866452 PMC12391555

