# Supplementary material for: Optimization of glucocorticoid administration in patients with sepsis using reinforcement learning: a multicenter retrospective study
Source: Front Cell Infect Microbiol. 2026 Aug 19;16:1856196. doi: 10.3389/fcimb.2026.1856196 (PMC13533693; doi:10.3389/fcimb.2026.1856196)
Supplement: Supplementary file 1 [file DataSheet1.docx]

Supplementary Material

**Supplementary Table1. Clinical variables extracted from the databases.**

| Category | Variable name | |  |  |  |  |
| --- | --- | --- | --- | --- | --- | --- |
| Demographic | age |  | |  |  |  |
| Demographic | gender |  | |  |  |  |
| Demographic | weight |  | |  |  |  |
| Treatment-related | glucocorticoid use | | |  |  |  |
| Treatment-related | vasopressor status | | |  |  |  |
| Treatment-related | intravenous fluid volume | | | |  |  |
| Vital signs | heart rate (average) | | |  |  |  |
| Vital signs | systolic blood pressure (SBP) | | | |  |  |
| Vital signs | diastolic blood pressure (DBP) | | | |  |  |
| Vital signs | mean blood pressure (MBP) | | | |  |  |
| Vital signs | respiratory rate | | |  |  |  |
| Vital signs | temperature | | |  |  |  |
| Vital signs | peripheral oxygen saturation (SpO_2_) | | | | |  |
| Laboratory | blood urea nitrogen (BUN) | | | |  |  |
| Laboratory | creatinine |  | |  |  |  |
| Laboratory | glucose |  | |  |  |  |
| Laboratory | sodium |  | |  |  |  |
| Laboratory | potassium | | |  |  |  |
| Laboratory | hemoglobin | | |  |  |  |
| Laboratory | platelet count | | |  |  |  |
| Laboratory | white blood cell count (WBC) | | | |  |  |
| Laboratory | international normalized ratio (INR) | | | | |  |
| Laboratory | prothrombin time (PT) | | | |  |  |
| Laboratory | partial thromboplastin time (PTT) | | | | |  |
| Laboratory | pH |  | |  |  |  |
| Laboratory | base excess | | |  |  |  |
| Severity scores | Glasgow Coma Scale (GCS) score | | | | |  |
| Severity scores | Sequential Organ Failure Assessment (SOFA) score | | | | | |
| Severity scores | Richmond Agitation-Sedation Scale (RASS) score | | | | | |
| Outcome | in-hospital mortality | | |  |  |  |

**Supplementary Figure 1.** Distribution of clinical variables before and after imputation. Kernel density plots comparing the distributions of selected variables in the original dataset (blue) and after multiple imputation (red). The overall distributions remained largely consistent after imputation.

**Supplementary Figure 2.** Results of LASSO regression for feature selection. (A) Ten-fold cross-validation for λ selection. Red dots indicate mean binomial deviance, and error bars represent ±1 standard error. Dashed lines denote the optimal λ values. (B) Coefficient profiles of candidate variables across log(λ).

**Supplementary Figure 3.** SHAP analysis of feature importance and contribution patterns under the CQL and clinician policies. (A–B) Feature importance ranked by mean absolute SHAP values. (C–D) SHAP summary plots illustrating the direction and magnitude of feature effects.

**Supplementary Figure 4.** Exploratory subgroup analysis of CQL policy value. Points show the mean FQE value difference between the CQL and clinician policies, and error bars indicate 95% confidence intervals.
